# In search of potential predictors of erythropoiesis-stimulating agents (ESAs) hyporesponsiveness: a population-based study

**DOI:** 10.1186/s12882-019-1554-0

**Published:** 2019-09-14

**Authors:** Ingrasciotta Ylenia, Lacava Viviana, Marcianò Ilaria, Giorgianni Francesco, Tripepi Giovanni, D’ Arrigo Graziella, Chinellato Alessandro, Tari Daniele Ugo, Santoro Domenico, Trifirò Gianluca

**Affiliations:** 1 Department of Biomedical and Dental Sciences and Morphofunctional Imaging, University of Messina, Via Consolare Valeria 1, Messina, Italy; 2 Dialysis and Nephrology Unit, University of Messina, Messina, Italy; 3 Unit of Clinical Pharmacology, A.O.U. Policlinico “G. Martino”, Messina, Italy; 4 CNR-IFC, Center of Clinical Physiology, Clinical Epidemiology of Renal Diseases and Hypertension, Reggio Calabria, Italy; 5 Pharmaceutical Service, Treviso Local Health Unit, Treviso, Italy; 6 Caserta Local Health Unit, Caserta, Italy; 7 Department of Medical Informatics, Erasmus Medical Center, Rotterdam, Netherlands

**Keywords:** Hyporesponsiveness, Erythropoiesis-stimulating agents, Prediction, Claims database

## Abstract

**Background:**

Evidences show that around 20% of biosimilar or originator erythropoiesis-stimulating agents (ESAs) users are hyporesponsive. Controversial post-marketing data exist on the predictors of ESA hyporesponsiveness. The aim of this study was to identify predictors of ESA hyporesponsiveness in patients with chronic kidney disease (CKD) or cancer in clinical practice.

**Methods:**

During the years 2009–2015, a multi-center, population-based, cohort study was conducted using claims databases of Treviso and Caserta Local Health Units (LHUs). All incident ESA users were characterized at baseline and the differences between the baseline hemoglobin (Hb) value, that is the Hb registered within 30 days prior to the first ESA dispensing (index date, ID) and each outcome Hb value (registered between 30 and 180 days after ID) were calculated and defined as delta Hb (ΔHb). Incident ESA users were defined as hyporesponsive if, during follow-up, they registered at least one ΔHb < 0 g/dL. Including all potential predictors of ESA hyporesponsiveness and stratifying by indication for use, univariate and multivariate binary logistic regression models and Receiver Operating Characteristic (ROC) curves were carried out.

**Results:**

In general, 1080 incident ESA users (CKD: 57.0%; cancer: 43.0%) were identified. In CKD, predictors of ESA hyporesponsiveness were C-reactive protein (OR = 1.2, 95% CI: 1.0–1.5; *P*-value = 0.060) and high levels of baseline Hb (OR = 1.7, 95% CI: 1.2–2.2; *P*-value< 0,001), the latter being also predictor of ESA hyporesponsiveness in cancer (OR = 1.7, 95% CI: 1.1–2.4; *P*-value = 0.007). Both in CKD and in cancer, the type of ESA, biosimilar or originator, was not a predictor of ESA hyporesponsiveness. In CKD, concomitant use of iron preparations (OR = 0.3, 95% CI: 0.2–0.7; *P*-value = 0.002) and of high dosage of angiotensin-converting enzyme inhibitors/angiotensin II-receptor blockers (OR = 0.5, 95% CI: 0.3–0.9; *P*-value = 0.022) were protective factors against ESA hyporesponsiveness.

**Conclusions:**

The study confirmed traditional potential predictors of hyporesponsiveness to ESA. The use of biosimilar or originator ESA was not a predictor of hyporesponsiveness in an outpatient setting from two large Italian areas. A better knowledge of the predictors of ESA response would allow a better anemia management to improve patients’ quality of life.

## Introduction

Erythropoiesis-stimulating agents (ESAs) are biological products, analogues of human erythropoietin, produced by cell lines using the recombinant DNA technology. ESAs are approved for the treatment of anaemia related to chronic kidney disease (CKD) or chemotherapy-induced in cancer patients. According to the Italian Medicines Agency, ESAs are indicated when hemoglobin (Hb) levels are lower than 11 g/dl in CKD patients and lower than 10 g/dl in cancer patients. In Italy, for both indications, haemoglobinemia has to range between 11 and 12 g/dl [1], avoiding a rise in Hb values greater than 2 g/dl over a four-week period.

Generally, the term “ESA hyporesponsive” refers to patients who need high doses of ESAs (25–100% higher doses than what recommended) to increase and/or maintain their Hb levels within the acceptable range [2]. More specifically, the Kidney Disease Outcomes Quality Initiative (KDOQI) guidelines define patients as ESA hyporesponsive if they do not experience an increase in Hb levels within the first month of ESA treatment, using an appropriate weight-based dosing (not graded) [3].

ESA hyporesponsiveness could be acute or chronic, but, to date, there is neither consensus nor shared position on the definition of the chronic condition in particular [3]. Based on the definition gave by *Sibbel* et al.*, “4 months of continuous ESA hyporesponsiveness [defined considering both Hb concentrations and ESAs doses] can be used to differentiate acute from chronic hyporesponsiveness”* [4].

A previously published population-based study, conducted on Italian administrative healthcare databases, evaluated the comparative effectiveness of both biosimilar and originator ESAs in CKD and cancer patients. Results highlighted that, in clinical practice, around 20% of ESA users were non-responders, defined as subjects experiencing no variations or a reduction in Hb levels within the first 3 months of ESA treatment. Furthermore, no differences were observed between different type of ESAs (i.e., biosimilars or originators), in terms of ESA responsiveness [5].

In patients with conservative end-stage renal disease, as well as in dialysis patients, ESA hyporesponsiveness and Hb level variability may lead to cardiovascular complications, increasing the risk of all-cause mortality, due to the required higher doses of ESA [6–8].

In both CKD and cancer patients, several factors may contribute to ESA hyporesponsiveness, such as iron deficiency, inflammation and malnutrition status, while chronic hyperparathyroidism may affect ESA response in CKD patients, specifically [9, 10].

Debate is still on-going regarding the potential effects of renin-angiotensin system inhibitors, such as angiotensin-converting enzyme (ACE) inhibitors or angiotensin II-receptor antagonists (ARBs), on the development of anaemia in patients with renal disease [11].

This naturalistic population-based study was aimed at identifying which factors could be associated to ESA hyporesponsiveness in anaemic patients with CKD or cancer, in the general population of two Italian Local Health Units (LHUs).

## Methods

### Data source

A population-based, retrospective, cohort study was conducted. As data source, claims databases of Treviso and Caserta LHUs, covering a total population of more than 1.5 million people during the years 2009–2015 (data were available till 2014 in Treviso LHU), were considered. Each prescription of ESA requires a specific therapeutic plan to be filled in by specialists, specifying the exact drug name, number of dispensed packages, dosing regimen and indication for use of the drug. These data can be linked, through anonymized patient unique identifier, to other claims databases including information on hospital discharge diagnoses, healthcare service payment exemptions, drug dispensing, outpatient diagnostic tests, results of laboratory tests (in Caserta LHU, these data are available only for a random sample of around 15% of the general population), etc. ICD-9-CM diagnosis codes were used to identify hospital discharge diagnoses and indications for use, while Anatomical Therapeutic Chemical (ATC) classification system codes and Italian marketing authorization (AIC) codes, which distinguish reference products from biosimilars and other ESAs still covered by patent, were used to identify drug dispensing. Additional details about data source can be found elsewhere [12].

### Study population

**Fig. 1:**
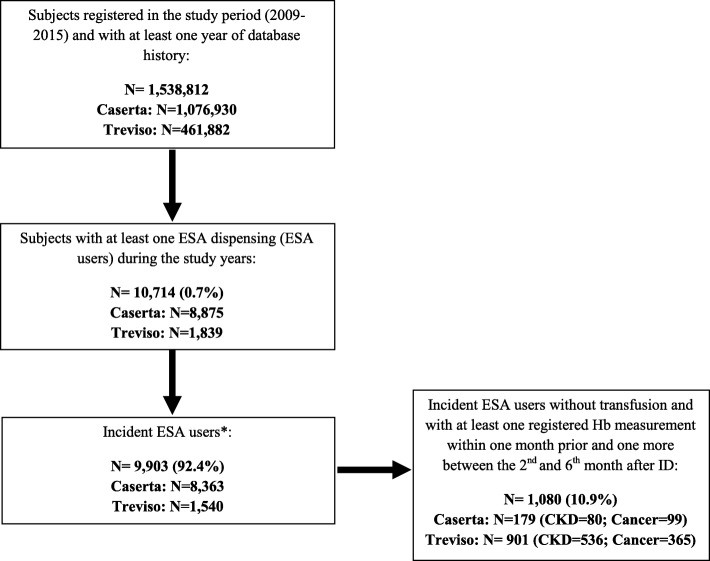
Flow-chart of study population. * no treatment within 6 months prior to Index Date (ID, i.e. date of ESA treatment start). ESA = erythropoiesis-stimulating agents; Hb = hemoglobin; CKD = chronic kidney disease

All the residents in Treviso or Caserta LHUs catchment areas in the years 2009–2015 were included in the study, if they had at least 1 year of database history, at least one ESA dispensing during the study period, with no ESAs dispensing within the previous 6 months (i.e. incident ESA users with 6-month washout period), at least one Hb measurement within 1 month prior to the date of the first ESA dispensing during the study period (i.e. Index Date, ID), defined as baseline Hb value, and at least another one between the 2nd and the 6th month after ID, defined as outcome Hb value (Fig. 1).

The included subjects were observed from the month prior to the ID to the first 6 months after the ID.

Patients were excluded in case they received at least one blood transfusion from 1 month prior to the ID to the last observed outcome Hb value.

### Study drugs

All the available ESAs in Italy during the study period were included in the study: epoetin alfa (ATC: B03XA01; Eprex®, Abseamed®, Binocrit®), epoetin beta (ATC: B03XA01; Neorecormon®), epoetin zeta (B03XA01; Retacrit®), darbepoetin alfa (ATC: B03XA02; Aranesp®), and methoxypolyethyleneglycol-epoetin beta (ATC: B03XA03; Mircera®). Binocrit®, Abseamed® and Retacrit® are biosimilars of the reference product (Eprex®), while all other ESAs are ESAs still covered by the patent.

### Data analysis

ESA users were categorized as CKD or cancer patients, according to indication for use recorded in the electronic therapeutic plan. In case of non-availability of electronic therapeutic plans, an algorithm described elsewhere was used to identify indication for use [12].

All incident ESA users were characterized at baseline, in terms of demographics, clinical parameters (e.g. hemoglobinemia and hematic level of creatinine, albumin, ferritin, folate, potassium, sideremia, parathyroid hormone, vitamin B_12_, C-reactive protein (CRP), and transferrin saturation), comorbidities including arrhythmia, ischemic heart diseases, diabetes mellitus, heart failure and hypertension, and concomitant use of iron preparations, folic acid, vitamin B_12_ and ACE inhibitors/ARBs.

During follow-up, the distribution of mean Hb values among incident ESA users was calculated.

The differences between the baseline Hb and each outcome Hb value were calculated and defined as delta Hb (ΔHb). Incident ESA users were classified as ESA hyporesponsive if, during follow-up, they registered at least one ΔHb < 0 g/dL. Only incident ESA users having a baseline Hb value lower than 11 g/dL were included in these analyses.

All analyses were stratified by indication for use.

### Sensitivity analysis

Due to the lack of a shared position on the description of ESA hyporesponsiveness, the definition of ESA hyporesponsiveness was modified in the sensitivity analyses. Incident ESA users with at least two consecutive outcome Hb values ≥11 g/dL, were classified as ESA responders, irrespective of ΔHb. Elsewhere, they were considered as ESA hyporesponsive patients.

### Statistical analysis

Depending on the distribution for quantitative variables, results were presented as mean ± standard deviation (SD) or median with interquartile range (IQR), and by absolute frequencies and percentages for categorical variables.

Univariate and multivariate binary logistic regression models were performed to identify predictors of ESA hyporesponsiveness, stratifying by indication for use (CKD and cancer). The dependent variable of the model was the hyporesponsiveness to ESA treatment, that is at least once ΔHb < 0 g/dL.

As covariates, all the potential predictors of ESA responsiveness identified from the database, including sex, age, baseline Hb value, ESA dosage at ID, type of ESA dispensed at ID (biosimilar, reference product or other ESAs still covered by patent), LHU, type of hospital discharge diagnosis (categorized into cardiovascular, non-cardiovascular or both cardiovascular and non-cardiovascular hospitalizations) within 1 year prior to ID, comorbidities (arrhythmia, ischemic heart disease, diabetes mellitus, heart failure, hypertension and dialysis, only for CKD patients), CKD stage or type of tumor (i.e. solid malignant, non-solid malignant, both solid and non-solid malignant, or not classified), concomitant drug use (e.g. iron preparations, vitamin B_12_, folic acid, high dosage of ACE inhibitor/ARBs) and laboratory values (e. g. hematic levels of creatinine, albumin, ferritin, folate, potassium, sideremia, parathyroid hormone, vitamin B12, C-reactive protein [CRP], transferrin saturation, acidosis) were included in the model. By restricting potential predictors to all those factors identified from the database, reduce the likelihood of an overstatement.

In the multivariate model, we included all the covariates, which were significantly associated to the outcome at the univariate analysis. For each model, a Receiver Operating Characteristic (ROC) curve was performed to predict the discriminatory power of the variables included in the model.

For each covariate tested as possible predictor of ESA hyporesponsiveness, the corresponding odds ratio (OR) were reported along with 95% confidence interval (95% CI).

All statistical analyses were performed using SAS 9.3 (SAS Institute, Cary, NC) and SPSS/PC, Version 21 (SPSS Inc., Chicago, Illinois, USA). The significance level for all statistical tests was set at *p*-value < 0.05.

## Results

On a total population of 1,538,812 subjects registered in Treviso and Caserta LHUs, 10,714 (0.7%) received at least one ESA dispensing during the years 2009–2015; of these, 1080 (10.1%) incident ESA users were included in the study, based on the above-mentioned inclusion criteria [CKD = 616 (57.0%); cancer = 464 (43.0%)] (Fig. 1).

As shown in Table 1, ESAs were in general more frequently used by males among CKD patients, and by females among cancer patients. Regarding age distribution, incident ESA users with CKD appeared to be on average older (mean age ± SD: 72.6 ± 14.7) than patients with cancer (66.9 ± 12.2). Although most of ESA users started ESA treatment having baseline Hb values within the range recommended by the Italian guidelines (Hb < 10 g/dL in cancer and Hb < 11 g/dL in CKD), 18.5% (*N* = 114) of CKD patients and 10.3% (*N* = 48) of cancer patients started ESA treatment with baseline Hb values ≥11 g/dL. Around 45% of incident ESA users received a biosimilar ESA at ID, irrespective of indication of use. In general, CKD patients were more likely to be hospitalized than cancer patients (66.2% vs. 56.2%), especially due to non-cardiovascular diseases. As compared to cancer patients, CKD patients were more likely to be affected by chronic comorbidities, such as hypertension (93.2% vs. 66.8%) and diabetes mellitus (41.9% of vs. 25.0%). Among ESA users with CKD, 410 (66.5%) were affected by stage IV-V CKD or were on dialysis. Instead, more than one third of cancer patients were affected by solid malignant neoplasms, although for most of cancer patients the type of tumor was not known (*N* = 208; 44.8%). CKD patients were more likely to be treated with iron preparations (CKD: 18.3%; cancer: 8.4%) or anti-hypertensive drugs (ACE inhibitor or ARBs) (CKD: 43.0%; cancer: 30.8%) than cancer patients. Considering laboratory parameters, no differences were found among cancer and CKD patients.

**Table 1:** Characterization of incident ESA users at baseline

	Cancer patients *N* = 464	CKD patients *N* = 616
Sex – N (%)		
Males	217 (46.8)	356 (57.8)
Females	247 (53.2)	260 (42.2)
Age – year^a^	66.9 ± 12.2	72.6 ± 14.7
Age category – N (%)		
< 45	22 (4.7)	38 (6.1)
45–64	154 (33.2)	126 (20.5)
65–79	227 (48.9)	234 (38.0)
≥ 80	61 (13.2)	218 (35.4)
Baseline Hb - g/dL^a^	9.7 ± 1.1	10.1 ± 1.1
Baseline Hb ≥11 g/dL - N (%)	48 (10.3)	114 (18.5)
Days of ESA exposure^a^	101.8 ± 40.5	119.4 ± 41.0
ESA dosage during the follow-up^a^		
IU	34,994.1 ± 9308.1	8564.6 ± 4835.4
Mcg	204.7 ± 132.1	49.9 ± 30.0
Catchment area –N (%)		
Caserta	99 (21.3)	80 (13.0)
Treviso	365 (78.7)	536 (87.0)
Type of ESA – N (%)		
Reference product	129 (27.8)	126 (20.5)
Biosimilar	209 (45.0)	284 (46.1)
Other ESAs covered by patent	126 (27.2)	206 (33.4)
Hospitalizations/PS visits – N(%)^b^		
No	203 (43.8)	208 (33.8)
Cardiovascular hosp.	6 (1.3)	44 (7.1)
Non cardiovascular hosp.	244 (52.6)	283 (45.9)
Both cardiovascular and non-cardiovascular hosp.	11 (2.4)	81 (13.1)
Comorbidities – N (%)^c^		
Arrhythmia	30 (6.5)	139 (22.6)
Ischemic heart disease	23 (5.0)	106 (17.2)
Diabetes mellitus	116 (25.0)	258 (41.9)
Heart failure	28 (6.0)	193 (31.3)
Hypertension	310 (66.8)	574 (93.2)
Dialysis	–	90 (14.6)
Stage of CKD – N(%)		
1 (GFR ≥ 90)	**–**	2 (0.3)
2 (90 > GFR ≥ 60)	**–**	11 (1.8)
3 (60 > GFR ≥ 30)	**–**	188 (30.5)
4 (30 > GFR ≥ 15)	**–**	230 (37.3)
5 and dialysis (GFR < 15 (or dialysis code))	**–**	180 (29.2)
Not classified		5 (0.8)
Type of tumor – N(%)		
Benign	4 (0.9)	**–**
Solid malignant	161 (34.7)	**–**
Non solid malignant	72 (15.5)	**–**
Both solid and non-solid malignant	19 (4.1)	**–**
Non classified	208 (44.8)	**–**
Concomitant drugs – N (%)^d^		
Iron preparations	39 (8.4)	113 (18.3)
Vitamin B_12_	7 (1.5)	12 (1.9)
Folic acid	37 (8.0)	59 (9.6)
ACE Inhibitors/ARBs	143 (30.8)	265 (43.0)
Laboratory values		
Albumin (g/dL; normal range: 3.5–5.5)^a^	3.6 ± 0.6	3.7 ± 0.6
Creatinine (mg/dL; normal range: M = 0.7–1.2; F = 0.6–1.2)^e^	0.9 (0.7–1.1)	2.5 (1.7–4.0)
Potassium (mEq/L; normal range: 3.6–5.0)^a^	4.4 ± 0.6	4.7 ± 0.7
Transferrin saturation (%)^a^	20.7 ± 14.3	22.3 ± 14.1
Sideremia (mcg/dL; normal range: M = 75–160; F = 60–150)^e^	56.0 (37.0–83.7)	50.0 (33.0–71.0)
Ferritin (mcg/L; normal range: M = 60–300; F = 30–150)^e^	278.4 (112.0–583.6)	150.6 (59.5–329.3)
Parathyroid hormone (pg/ml; normal range: 10–60)^e^	47.0 (27.0–79.0)	160.0 (85.0–300.3)
Vitamin B_12_ (ng/ml; normal range:300–900)^a^	491.6 ± 224.3	506.9 ± 248.4
Folate (ng/ml; normal range: 2.7–17)^e^	6.0 (3.9–8.8)	5.2 (3.5–7.6)
CRP (mg/dL; normal value: < 0.5)^e^	0.9 (0.3–4.4)	0.9 (0.3–3.1)

*CKD* Chronic kidney disease, *GFR* Glomerular filtration rate, *ACE* angiotensin converting enzyme, *ARBs* Angiotensin II receptor antagonists, *CRP* C-reactive protein, *SD* Standard deviation, *IQR* Interquartile range, *IU* International Unit, *Mcg* Microgram

^a^Data are expressed as mean ± SD

^b^Evaluated within the year prior to ID

^c^Evaluated any time prior to ID

^d^Evaluated within 3 months prior to ID

^e^Data are expressed as median and IQR

**Fig. 2:**
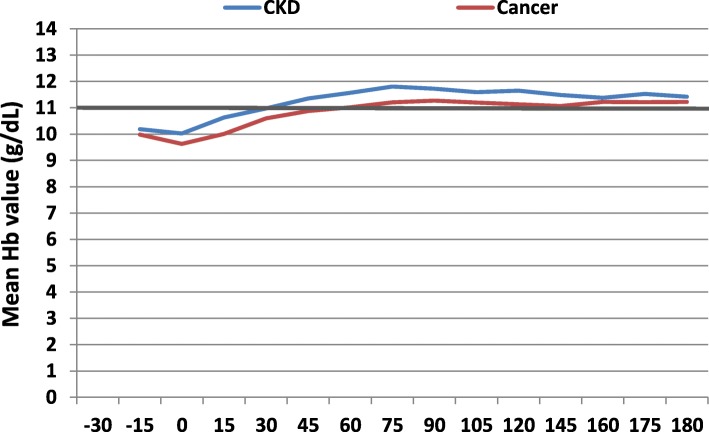
Mean Hb variation during the follow-up, stratified by indication for use

The target Hb value, as recommended by the Italian Medicines Agency, was reached on average between 45 and 60 days after ID and was thereafter stable during follow-up (Fig. 2).

Excluding incident ESA users with baseline Hb values higher than recommended (i.e., ≥11 g/dL), we observed that most of subjects included in the study cohort reached, at least once, the target Hb values (11 ≤ Hb levels≤12 g/dL), according to recommendations from Italian guidelines, despite 664 (61.5%) incident ESA users reached Hb levels> 13.0 g/dL, at least once during follow-up (Additional file 1).

**Fig. 3:**
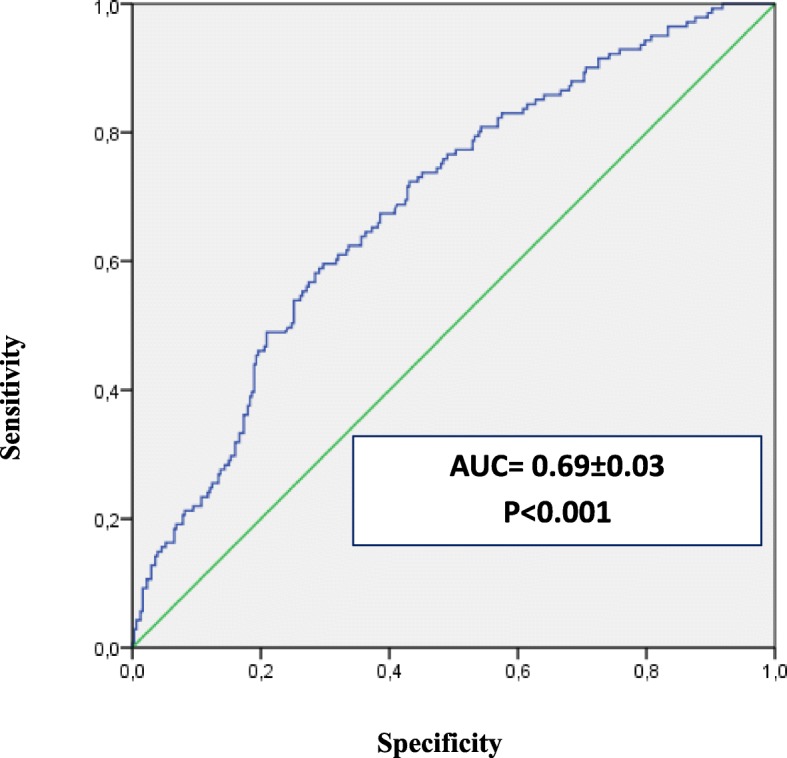
ROC curve to predict the discriminant power of non-responsiveness in CKD

**Fig. 4:**
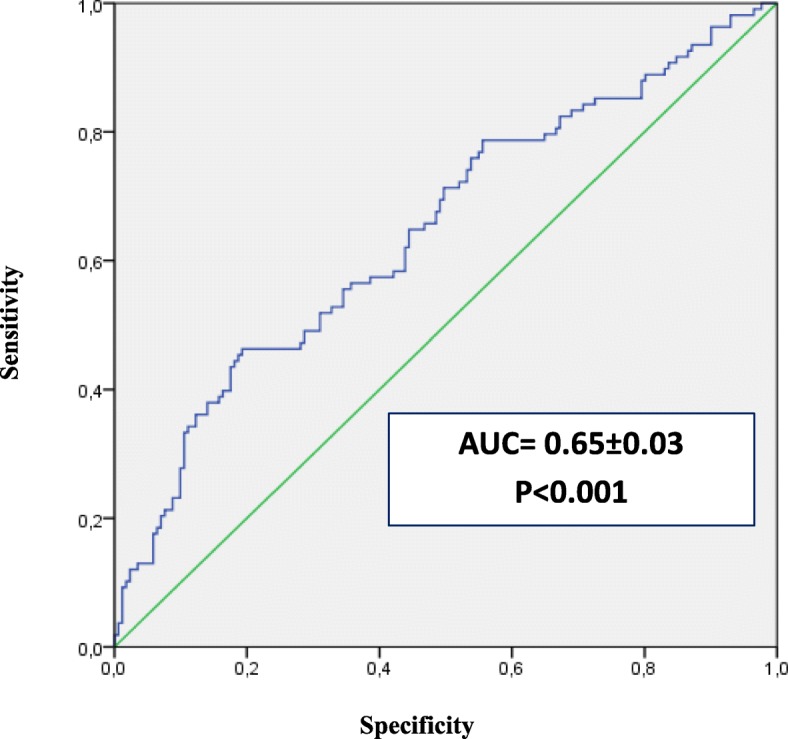
ROC curve to predict the discriminant power of non-responsiveness in cancer

Table 2 showed that, for each cohort, the proportion of ESA hyporesponsive patients was similar using the two approaches of ESA hyporesponsiveness. According to the given definition of ESA hyporesponsiveness, the multivariate binary logistic regression showed that the type of dispensed ESA (biosimilar or originator) was not a predictor of ESA response in CKD. Moreover, high baseline Hb values (OR = 1.7, 95% CI: 1.2–2.2; *P*-value< 0.001) and CRP hematic levels (OR = 1.2, 95% CI: 1.0–1.5; *P*-value = 0.060) were associated to ESA hyporesponsiveness in CKD (Table 3), while high baseline Hb values (OR = 1.7, 95% CI: 1.1–2.4; *P*-value = 0.007) and prior ischemic heart disease diagnosis (OR = 2.7, 95% CI: 0.9–7.9; *P*-value = 0.072) were predictors of ESA hyporesponsiveness in cancer patients (Table 4). On the contrary, ESA hyporesponsiveness was decreased by concomitant use of iron preparations (OR = 0.3, 95% CI: 0.2–0.7; *P*-value = 0.002) and high dosage of ACE inhibitors/ARBs (OR = 0.5, 95% CI: 0.3–0.9; *P*-value = 0.022) in CKD patients and by higher levels of albumin and potassium in cancer patients, although not significantly (*P*-values> 0.005). The discriminatory power of the predictive response of the variables included into the models was good, as confirmed by the ROC curves (Figs. 3-4).

**Table 2:** Frequency of incident ESA hyporesponders

	Cancer *N* = 416 (%)	CKD *N* = 502 (%)
Non responsiveness		
ΔHb < 0 g/dL^a^	146 (35.1)	152 (30.3)
Hb < 11 g/dL^b^	135 (32.4)	147 (29.3)

Only incident ESA users having a baseline Hb value lower than 11 g/dL were included in these analyses

^a^Incident ESA users with at least one ΔHb < 0 g/dL

^b^Incident ESA users with Hb values < 11 g/dL or with only one Hb value ≥11 g/dL registered between the 2nd and the 6th month after ID

**Table 3:** Multivariate binary logistic regression to evaluate non responsiveness to ESAs between the 2nd and the 6th month after ID in CKD patients

	Non responsiveness ΔHb < 0 g/dL (at least once)	
	OR (95% CI)	*P*-value
Baseline Hb (g/dL)	1.7 (1.2–2.2)	< 0.001
Comorbidities		
Hypertension	0.8 (0.3–1.7)	0.513
Concomitant drugs		
Iron preparations	0.3 (0.2–0.7)	0.002
Folic acid	0.5 (0.2–1.1)	0.100
High dosage ACE inhibitors/ARBs	0.5 (0.3–0.9)	0.022
Laboratory Values		
CRP	1.2 (1.0–1.5)	0.060

Transferrin saturation covariate was excluded because of the high proportion of missing values (> 50%)

CKD ESA users starting the treatment at baseline Hb ≥ 11 g/dL were excluded

*ACE* angiotensin converting enzyme, *CRP* C-reactive protein

**Table 4:** Multivariate binary logistic regression to evaluate non responsiveness to ESAs between the 2nd and the 6th month after ID in cancer patients

	Non responsiveness ΔHb < 0 g/dL (at least once)	
	OR (95% CI)	*P*-value
Baseline Hb (g/dL)	1.7 (1.1–2.4)	0.007
Comorbidities		
Ischemic heart disease	2.7 (0.9–7.9)	0.072
Laboratory Values		
Albumin (g/dL)	0.7 (0.5–1.1)	0.091
Potassium (mEq/L)	0.7 (0.4–1.0)	0.063
CRP (mg/dL)	1.1 (0.9–1.3)	0.537

Covariates as ferritin and vitamin B_12_ levels were excluded because of the high proportion of missing values (> 40%)

Cancer ESA users starting the treatment at baseline Hb ≥ 11 g/dL were excluded

By modifying the ESA hyporesponsiveness definition in the sensitivity analysis, high baseline Hb value was a positive predictor of responsiveness both in CKD (OR = 0.7, 95% CI: 0.5–1.0; *P*-value = 0.053) and in cancer patients (OR = 0.5, 95% CI: 0.3–0.8; *P*-value = 0.003); that is, patients with high baseline Hb values had more chance to reach the target Hb values rather than patients starting ESA treatment with low baseline Hb values (Additional files 2, 3, 4, 5). In addition, concomitant use of iron preparations and acidosis condition increased ESA response in CKD patients, with a good predictive power (AUC = 0.6969 ± 0.03; *P*-value< 0.001). Moreover, males with CKD (OR = 0.5, 95% CI: 0.3–0.9; *P*-value: 0.011) seemed to be more responsive than females.

## Discussion

Anaemia is a common complication in both cancer and CKD patients and it could contribute to a poor prognosis. ESA therapy represents the main treatment to increase Hb levels in such groups of patients, leading to improvement of quality of life and reducing the risk of cardio- and cerebrovascular complications, as well as the requirement of blood transfusions. However, ESA therapy must be carefully handled due to the increased risk of stroke in older patients having Hb levels above the target range. Indeed, due to the occurrence of ESA resistance, the need for higher doses of ESA may increase the risk of developing cardiovascular diseases and, ultimately, death [13]. Moreover, *Minutolo* et al. demonstrated that ESA hyporesponsiveness increased the risk of end stage renal disease by 2.5-fold in CKD patients [14].

Our data confirmed that the inflammatory condition and the iron intake affect ESA response. Inflammatory cytokines may affect the development of anemia through suppression of bone marrow erythropoiesis, suppression of erythropoietin production, or interfering with the iron status [15]. Several published studies demonstrated that high levels of CRP in hemodialysis patients were associated with ESA hyporesponsiveness, leading to an increased risk of death [16–18]. Although the prevalence of ESA hyporesponsiveness in hemodialysis patients is similar to that found in non-dialysis patients, limited studies on the predictors of ESA hyporesponsiveness have been conducted in the latter population [14].

Regarding the iron intake, our results highlighted that the use of iron preparations was a predictive factor of ESA response, whilst serum iron and ferritin were not independently associated to responsiveness to ESA treatment. Although previous studies on hemodialysis patients demonstrated that an altered iron status (in terms of low transferrin saturation levels and/or low ferritin levels), is a common factor inducing ESA hyporesponsiveness [19], there is no general consensus regarding the role of iron status as a predictor of ESA response. A recent study examined the relationship between iron markers, such as transferrin saturation and ferritin levels, and ESA responsiveness. Finding from the study highlighted that transferrin saturation, but not ferritin, was statistically associated to ESA hyporesponsiveness [20]. On the hand, in our study, transferrin saturation covariate was excluded from the analysis, due to the high proportion of missing values (> 50%).

*Minutolo* et al. studied for the first time the risk of end-stage renal disease in CKD patients, who were hyporesponsive to ESA treatment. The study findings demonstrated that ESA hyporesponsiveness correlated to an increased risk of end-stage renal disease and the authors suggested that high ESA doses, together with the persistence of anemia, could lead to hypoxia, tubular atrophy and interstitial fibrosis, thus causing the progression of the renal damage. On the other hand, no correlation between the iron markers, CRP levels, serum parathyroid hormone, body mass index and ESA response was found [14].

The influence of gender on ESA response is still controversial. Female gender was associated with ESA hyporesponsiveness in our study cohort. This result is in line with previous studies [19, 21], and may be related to the underlying differences in iron release from reticuloendothelial cells between the two genders [22]. Conversely, other studies demonstrated that males were more likely to be ESA hyporesponsive, in comparison to women [23, 24].

In our study, high doses of ACE inhibitor and/or ARBs were related to ESA responsiveness. This data has been controversially discussed in previous papers. Several studies showing that ACE inhibitors and ARBs are associated to an increase of ESA hyporesponsiveness [23, 25] hypothesized that these anti-hypertensive drugs may interfere with erythropoiesis. It is known that the activation of renin-angiotensin system enhances the erythropoietin productions [26], while its inhibition due to ACE inhibitors may exacerbate anaemia [27]. Moreover, it has been demonstrated that ACE inhibitors may cause an increase in serum N-acetyl-seryl-aspartyl-lysyl-proline (Ac-SDKP) levels, which inhibit the recruitment of pluripotent erythroid cells in bone marrow [28]. Other potential mechanisms by which the considered anti-hypertensive drugs may cause anemia are the serum reduction of specific cytokines, such as interleukin-12, and/or of insulin-like growth factor-1, which physiologically stimulate erythropoiesis [11].

Our results also demonstrated that ESA users with metabolic acidosis (pH < 7.38 and serum HCO^3−^ < 22 mmol/l) had a good ESA response. Due to the lack of evidence explaining such potential association between metabolic acidosis and ESA hyporesponsiveness, further investigations on this potential predictive factor are needed.

Considering cancer patients, we found that higher baseline Hb values were associated with ESA hyporesponsiveness (*p*-value = 0.007), together with the history of ischemic heart disease, although this correlation is close to be significant (*p*-value = 0.072). The role of cardiovascular diseases as predictors of ESA hyporesponsiveness has been previously studied [18, 29] in CKD patients and the most liable mechanism is related to an increased production of inflammatory cytokines, such as interleukins 1 and 6, Tumor Necrosis Factor and interferon, which induce apoptosis in erythroid progenitor cells and decrease the iron availability by stimulating hepcidin production [30]. Further analyses are, on the other hand, required to confirm the role of cardiovascular diseases as predictors of ESA hyporesponsiveness in cancer setting.

## Strengths and limitations

This study has several strengths. Firstly, we may explore data on ESA dispensing from two large Italian LHUs over a 7-year observation period. Secondly, thanks to the electronic therapeutic plans, information on the exact brand name, number of dispensed packages, and indication for use were available. Moreover, we could explore variations in Hb values as a result of ESA treatment, using real-world data from more than 1000 ESA users. Most of the previous randomized clinical trials were conducted considering CKD patients only, while our study explored the potential predictors of ESA hyporesponsiveness in both CKD and cancer patients. However, some limitations warrant caution. The high frequency of missing values for some variables considered into the study (namely: transferrin saturation for CKD, as well as ferritin and vitamin B12 for cancer) precluded the possibility to test the independent effect of these risk factors on the study outcome. Thus such an issue remains to be investigated in a specifically designed future cohort study. Furthermore, although we tested into the models a series of laboratory risk factors assessed proximally to the Hb measurement, the possibility of residual time dependent confounding due to unmeasured confounders cannot be excluded. Some ESA as well as concomitant drugs (i.e. iron preparations) dispensing might not have been fully captured by the LHUs databases, as these drugs may be initially dispensed directly by the public hospitals or purchased by patients as out of pocket, thus not being traced using the study data sources. However, it is unlikely that this limitation affected the study results, as the potential selection bias is expected to be minimal and non-differential between ESAs responders and hyporesponders.

Finally, since the exact body weight of each ESA user was not available and we could not evaluate the exact ESA dosing regimen, we defined ESA hyporesponsiveness as a decrease in Hb levels and, in the sensitivity analysis, as the failure in achieving Hb values ≥11 g/dL, as reported by *Suttorp* et al. in a multi-center, prospective study [7].

.

## Conclusions

This study tries to identify some potential predictive factors associated with ESA hyporesponsiveness. Covariates as serum CRP or high levels of baseline Hb were confirmed to be associated with poor response to ESA. A better knowledge of the factors associated with ESA response may help avoiding the use of higher ESA doses, and allow a better anaemia management in order to improve the patients’ quality of life and reduce morbidity and mortality of both CKD and cancer patients.

## Supplementary information

**Additional file 1.** Distribution of Hb values during the follow-up among incident ESA users, stratified by indication for use: a) CKD; b) Cancer.

**Additional file 2.** Multivariate binary logistic regression to evaluate non responsiveness to ESAs between the 2nd and the 6th month after ID in CKD patients.

**Additional file 3.** ROC curve to predict the discriminant power of non-responsiveness in CKD.

**Additional file 4.** Multivariate binary logistic regression to evaluate non responsiveness to ESAs between the 2nd and the 6th month after ID in cancer patients.

**Additional file 5.** ROC curve to predict the discriminant power of non-responsiveness in Cancer.

## Data Availability

The data that support the findings of this study are available from Caserta and Treviso LHUs, but restrictions apply to the availability of these data, which were used under license for the current study, and so are not publicly available. Data are however available from the authors upon reasonable request and with permission of Caserta and Treviso LHU.

## Abbreviations

ACE: Angiotensin-converting enzyme inhibitors
AIC code: Italian marketing authorization code
ARBs: Angiotensin II-receptor antagonists
ATC code: Anatomical Therapeutic Chemical code
CI: Confidence interval
CKD: Chronic kidney disease
CRP: C-reactive protein
ESAs: Erythropoiesis-stimulating agents
GFR: Glomerular filtration rate
Hb: Hemoglobin
ICD9-CM code: International Classification of Disease, 9th revision, clinical modification code
ID: Index date
IQR: Interquartile range
IU: International Unit
KDOQI: Kidney Disease Outcomes Quality Initiative
LHUs: Local Health Units
Mcg: Microgram
OR: Odds ratio
ROC curves: Receiver Operating Characteristic curves
SD: Standard deviation
