# Correction to: In search of potential predictors of erythropoiesis-stimulating agents (ESAs) hyporesponsiveness: a population-based study

**DOI:** 10.1186/s12882-020-01891-w

**Published:** 2020-07-09

**Authors:** Ylenia Ingrasciotta, Viviana Lacava, Ilaria Marcianò, Francesco Giorgianni, Giovanni Tripepi, Graziella D’. Arrigo, Alessandro Chinellato, Daniele Ugo Tari, Domenico Santoro, Gianluca Trifirò

**Affiliations:** 1 Department of Biomedical and Dental Sciences and Morphofunctional Imaging, University of Messina, Via Consolare Valeria 1, Messina, Italy; 2 Dialysis and Nephrology Unit, University of Messina, Messina, Italy; 3 Unit of Clinical Pharmacology, A.O.U. Policlinico “G. Martino”, Messina, Italy; 4 CNR-IFC, Center of Clinical Physiology, Clinical Epidemiology of Renal Diseases and Hypertension, Reggio Calabria, Italy; 5 Pharmaceutical Service, Treviso Local Health Unit, Treviso, Italy; 6 Caserta Local Health Unit, Caserta, Italy; 7 Department of Medical Informatics, Erasmus Medical Center, Rotterdam, Netherlands

**Correction to: BMC Nephrology (2019) 20:359**

**https://doi.org/10.1186/s12882-019-1554-0**

Following publication of the original article [1], the authors identified that the first name and the last name of all the authors were interchanged.

Incorrect: Ingrasciotta Ylenia1*†, Lacava Viviana2†, Marcianò Ilaria3, Giorgianni Francesco3, Tripepi Giovanni4, D’ Arrigo Graziella4, Chinellato Alessandro5, Tari Daniele Ugo6, Santoro Domenico2 and Trifirò Gianluca1,3,7

Correct: Ylenia Ingrasciotta 1*†, Viviana Lacava 2†, Ilaria Marcianò 3, Francesco Giorgianni 3, Giovanni Tripepi 4, Graziella D’ Arrigo4, Alessandro Chinellato 5, Daniele Ugo Tari6, Domenico Santoro 2 and Gianluca Trifirò 1,3,7

The author group has been updated above and the original article [1] has been corrected.
