# Supplementary material for: In search of potential predictors of erythropoiesis-stimulating agents (ESAs) hyporesponsiveness: a population-based study
Source: BMC Nephrol. 2019 Sep 14;20:359. doi: 10.1186/s12882-019-1554-0 (PMC6744676; doi:10.1186/s12882-019-1554-0)
Supplement: Supplementary file 1 — Additional file 1. Distribution of Hb values during the follow-up among incident ESA users, stratified by indication for use: a) CKD; b) Cancer. [file 12882_2019_1554_MOESM1_ESM.pdf]

Additional file 1. Distribution of Hb values during the follow-up among incident ESA users, stratified by indication for use: a) CKD; b) Cancer

a) CKD

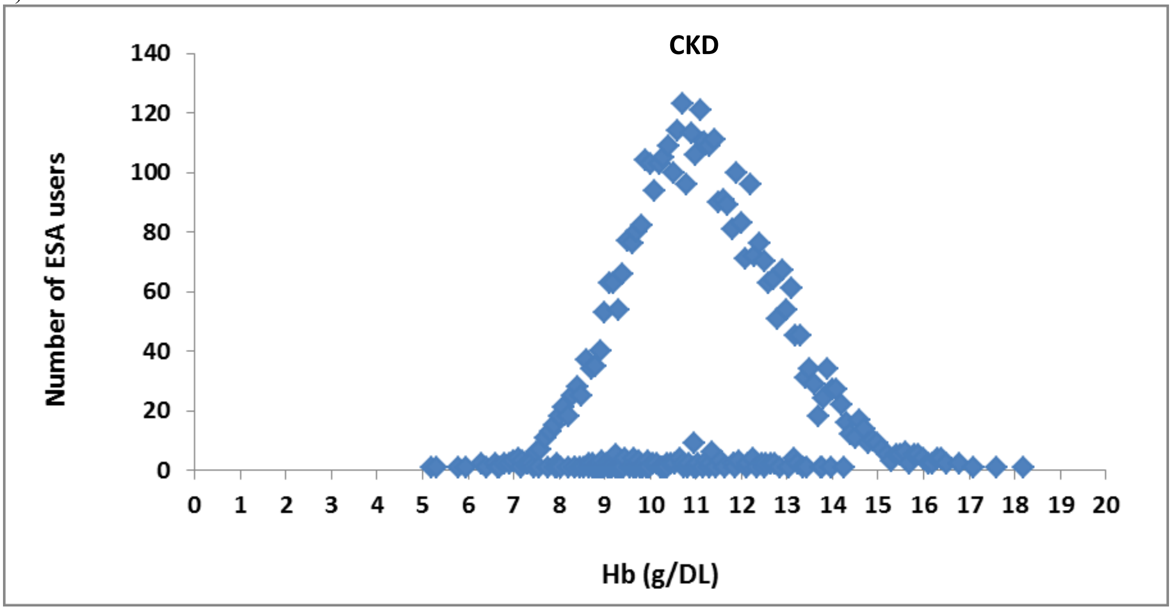

b) Cancer

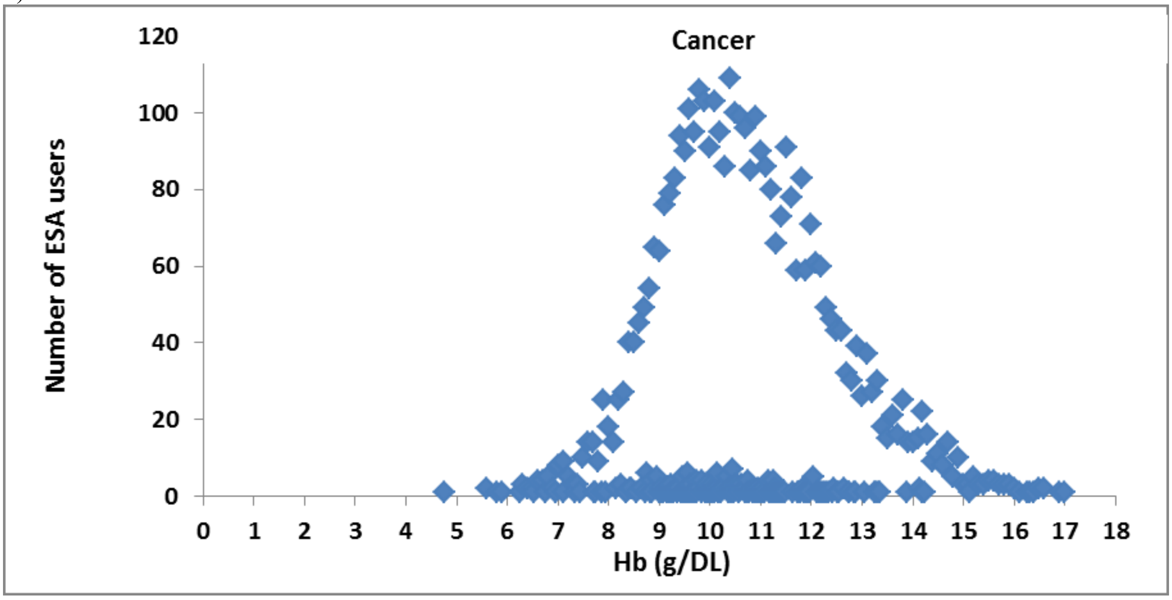

Incident ESA users starting the treatment at baseline Hb≥11 g/dL were excluded
