# Supplementary material for: In search of potential predictors of erythropoiesis-stimulating agents (ESAs) hyporesponsiveness: a population-based study
Source: BMC Nephrol. 2019 Sep 14;20:359. doi: 10.1186/s12882-019-1554-0 (PMC6744676; doi:10.1186/s12882-019-1554-0)
Supplement: Supplementary file 2 — Additional file 2. Multivariate binary logistic regression to evaluate non responsiveness to ESAs between the 2nd and the 6th month after ID in CKD patients. [file 12882_2019_1554_MOESM2_ESM.pdf]

Additional file 2. Multivariate binary logistic regression to evaluate non responsiveness to ESAs between the 2nd and the 6th month after ID in CKD patients

	**Non responsiveness (Hb<11g/dL) N=147**	
	**OR (95% CI)**	**P-value**
**Age (1 year)**	1.0 (1.0-1.1)	0.161
**Sex (Males)**	**0.5 (0.3-0.9)**	**0.011**
**Baseline Hb - g/dL**	**0.7 (0.5-1.0)**	**0.053**
**ESA type**		
**Reference product**	**Reference**	
Biosimilar	1.7 (0.8-3.5)	0.142
Other ESAs covered by patent	1.4 (0.7-2.8)	0.392
**Comorbidities**		
Diabetes mellitus	1.4 (0.8-2.2)	0.188
Heart failure	1.4 (0.8-2.4)	0.236
**Concomitant drugs**		
Iron preparations	**0.4 (0.2-0.8)**	**0.008**
High dosage ACE inhibitors/ARBs	1.0 (0.6-1.8)	0.931
**Laboratory values**		
Albumin (g/dL)	0.9 (0.6-1.3)	0.503
Creatinine (mg/dL)	1.0 (0.9-1.1)	0.783
Acidosis	**0.6 (0.3-0.9)**	**0.037**

CKD ESA users with at least two consecutive Hb values ≥11 g/dL registered between the 2nd and the 6th month after ID were considered ESA responders
