# Supplementary figures and images for: In search of potential predictors of erythropoiesis-stimulating agents (ESAs) hyporesponsiveness: a population-based study
Source: BMC Nephrol. 2019 Sep 14;20:359. doi: 10.1186/s12882-019-1554-0 (PMC6744676; doi:10.1186/s12882-019-1554-0)

**Additional file 3.** ROC curve to predict the discriminant power of non-responsiveness in CKD

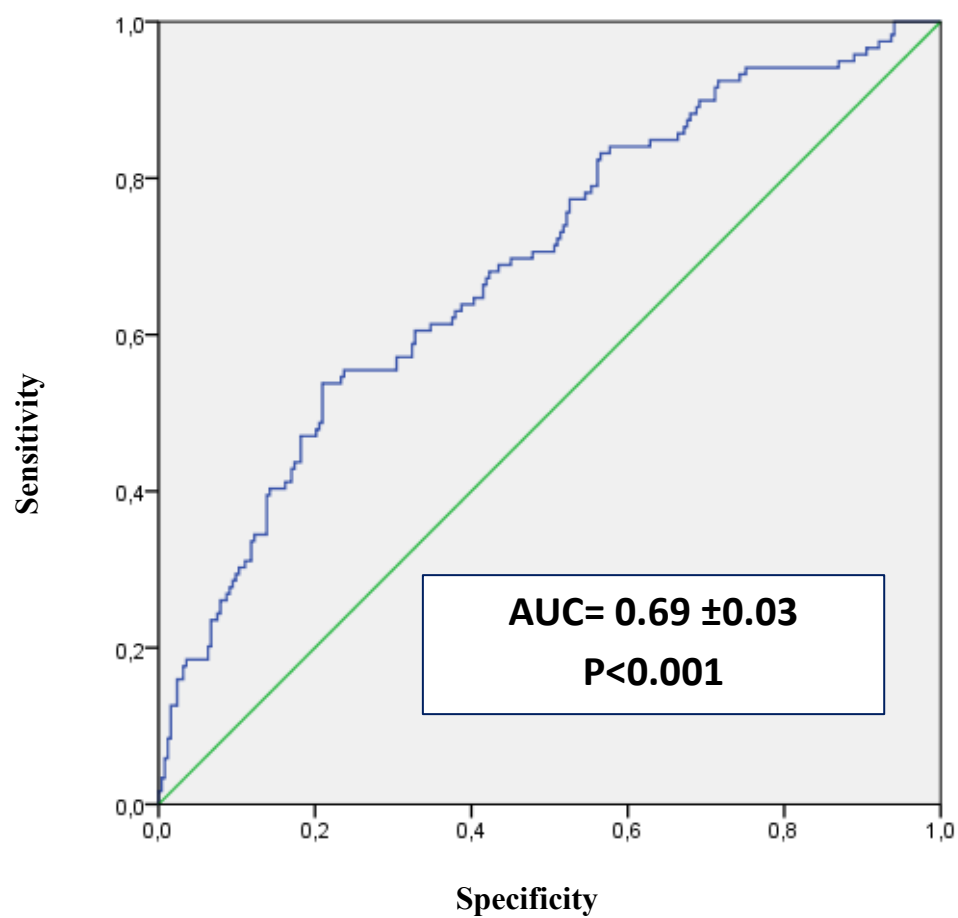

Supplement: Supplementary file 3 — Additional file 3. ROC curve to predict the discriminant power of non-responsiveness in CKD. [file 12882_2019_1554_MOESM3_ESM.pdf]

**Additional file 5.** ROC curve to predict the discriminant power of non-responsiveness in Cancer

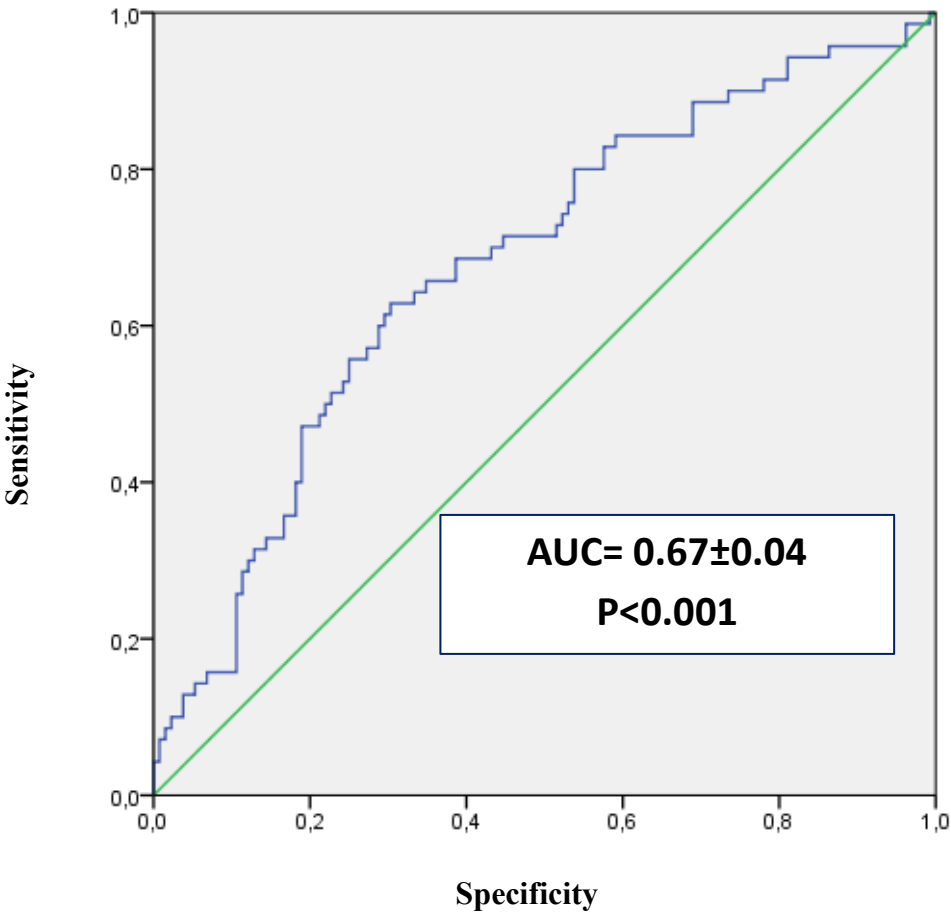

Supplement: Supplementary file 5 — Additional file 5. ROC curve to predict the discriminant power of non-responsiveness in Cancer. [file 12882_2019_1554_MOESM5_ESM.pdf]
