# Supplementary material for: In search of potential predictors of erythropoiesis-stimulating agents (ESAs) hyporesponsiveness: a population-based study
Source: BMC Nephrol. 2019 Sep 14;20:359. doi: 10.1186/s12882-019-1554-0 (PMC6744676; doi:10.1186/s12882-019-1554-0)
Supplement: Supplementary file 4 — Additional file 4. Multivariate binary logistic regression to evaluate non responsiveness to ESAs between the 2nd and the 6th month after ID in cancer patients. [file 12882_2019_1554_MOESM4_ESM.pdf]

Additional file 4. Multivariate binary logistic regression to evaluate non responsiveness to ESAs between the 2nd and the 6th month after ID in cancer patients

	**Non responsiveness (Hb<11g/dL) N=135**	
	**OR (95% CI)**	**P-value**
**Baseline Hb - g/dL**	**0.5 (0.3-0.8)**	**0.003**
**Acidosis**	0.6 (0.3-1.1)	0.109
**Concomitant drugs**		
High dosage ACE inhibitors/ARBs	1.6 (0.5-5.0)	0.391
**Laboratory values**		
Albumin (g/dL)	1.0 (0.6-1.6)	0.907
Ferritin (mcg/L)	1.0 (1.0-1.1)	0.233

Cancer ESA users with at least two consecutive Hb values ≥11 g/dL registered between the 2nd and the 6th month after ID were considered ESA responders

Vitamin B_{12} covariates were excluded because of the high proportion of missing values (>70%)
